# 1-Cyano­methyl-4-aza-1-azonia­bicyclo­[2.2.2]octane bromide dihydrate

**DOI:** 10.1107/S1600536810019926

**Published:** 2010-06-05

**Authors:** Ying Cai

**Affiliations:** a Ordered Matter Science Research Center, Southeast University, Nanjing 211189, People’s Republic of China

## Abstract

In the crystal structure of the title compound, C_8_H_14_N_3_^+^·Br^−^·2H_2_O, inter­molecular O—H⋯O and O—H⋯Br hydrogen bonding occurs. The water mol­ecules are connected into chains extending in the *a*-axis direction. The bromide anions are connected to the water mol­ecules, forming 10-membered rings. The cations are connected to the anions *via* weak C—H⋯Br inter­actions. Two carbon atoms of the cation are disordered and were refined using a split model (occupancy ratio 0.70:0.3).

## Related literature

For uses of DABCO (1,4-biaza­bicyclo­[2.2.2]octa­ne) and its derivatives, see: Basaviah *et al.* (2003 ▶); Chen *et al.* (2010 ▶). 
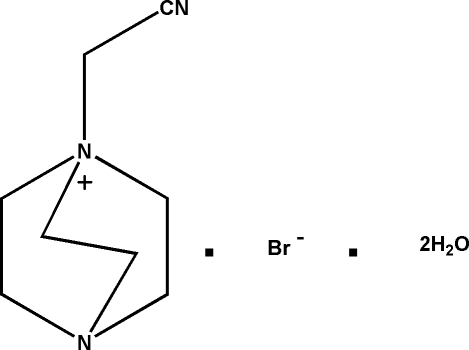


## Experimental

### 

#### Crystal data

C_8_H_14_N_3_^+^·Br^−^·2H_2_O
*M* *_r_* = 268.16
Orthorhombic, 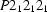

*a* = 7.461 (5) Å
*b* = 12.008 (7) Å
*c* = 13.236 (8) Å
*V* = 1185.8 (13) Å^3^
*Z* = 4
Mo *K*α radiation
μ = 3.45 mm^−1^
*T* = 293 K
0.20 × 0.20 × 0.20 mm

#### Data collection

Rigaku Mercury CCD diffractometer
Absorption correction: multi-scan (*CrystalClear*; Rigaku, 2005 ▶) *T*_min_ = 0.701, *T*_max_ = 1.000
13047 measured reflections
2711 independent reflections
2219 reflections with *I* > 2σ(*I*)
*R*_int_ = 0.073

#### Refinement

*R*[*F* ^2^ > 2σ(*F* ^2^)] = 0.041
*wR*(*F* ^2^) = 0.077
*S* = 1.01
2711 reflections
140 parameters
101 restraints
H-atom parameters constrained
Δρ_max_ = 0.29 e Å^−3^
Δρ_min_ = −0.29 e Å^−3^
Absolute structure: Flack (1983 ▶), 1134 Friedel pairs
Flack parameter: 0.033 (14)

### 

Data collection: *CrystalClear* (Rigaku, 2005 ▶); cell refinement: *CrystalClear*; data reduction: *CrystalClear*; program(s) used to solve structure: *SHELXS97* (Sheldrick, 2008 ▶); program(s) used to refine structure: *SHELXL97* (Sheldrick, 2008 ▶); molecular graphics: *SHELXTL* (Sheldrick, 2008 ▶); software used to prepare material for publication: *SHELXL97*.

## Supplementary Material

Crystal structure: contains datablocks I, global. DOI: 10.1107/S1600536810019926/nc2186sup1.cif

Structure factors: contains datablocks I. DOI: 10.1107/S1600536810019926/nc2186Isup2.hkl

Additional supplementary materials:  crystallographic information; 3D view; checkCIF report

## Figures and Tables

**Table 1:** Hydrogen-bond geometry (Å, °)

*D*—H⋯*A*	*D*—H	H⋯*A*	*D*⋯*A*	*D*—H⋯*A*
O1—H1*O*1⋯Br1	0.82	2.58	3.354 (3)	159
O2—H1*O*2⋯O1	0.82	1.98	2.791 (4)	170
O1—H2*O*1⋯O2^i^	0.82	1.99	2.788 (5)	164
O2—H2*O*2⋯Br1^i^	0.82	2.50	3.314 (3)	172
C7—H7*A*⋯Br1	0.97	2.81	3.740 (5)	161
C7—H7*B*⋯Br1^ii^	0.97	2.92	3.792 (8)	151

Symmetry codes: (i) 
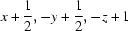
; (ii) 
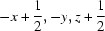
.

**Fig. 1.**
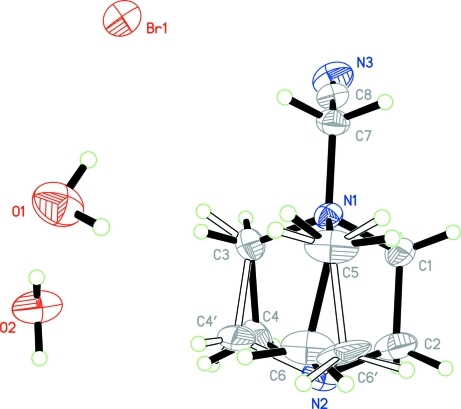
Crystal structure of the title compound with labelling and displacement ellipsoids drawn at the 30% probability level. Disordering is shown with full and open bonds.

**Fig. 2.**
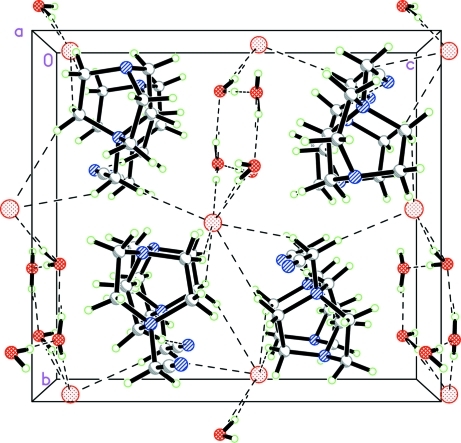
Crystal structure of the title compound with view along the a axis. Disordered C and H atoms are omitted and intermolecular hydrogen bonding is shown as dashed lines.

## supplementary crystallographic information

### Comment

1,4-Diazabicyclo[2.2.2]octane (DABCO) is used as a organocatalyst for a large number of reactions because of its nucleophilicity (Basaviah *et al.*, 2003) and some of its derivatives are ferroelectrics (Chen *et al.*, 2010). The structure determination of the title compound was performed within a project on the electric properties of 1,4-Diazabicyclo[2.2.2]octane derivatives. Within this project the crystals of the title compound were obtained by accident.

In the crystal stucture of the title compound two C atoms of the cation are disordered (Fig. 1). The cations and anions are connected by weak intermolecular C—H···Br interactions. The bromide anions are additionally linked to the water molecules *via* intermolecular O—H···Br hydrogen bonding and the water molecules are connected into chains that elongate in the direction of the *a* axis (Fig. 2). Each water molecule act as hydrogen bond donor and acceptor. The bromide anions and the water molecules forming ten-membered rings.

### Experimental

1,4-Diaza-bicyclo[2.2.2]octane (dabco) (0.05?mol, 5.6?g) and bromoacetonitrile (0.1?mol, 12.00?g) were dissolved in CH_3_CN(40?ml). The mixture was stirred for 1?h leading to a white precipitate of the title compound whish was filtered off, washed with acetonitrile and dried. Yield: 80%. Afterwards a mixture of 1-(cyanomethyl)-4-aza-1-azonia-bicyclo[2.2.2]octane bromide (0.01?mol 2.32?g) and tetrafluoro-borate sodium (0.01?mol 1.10?g) in H_2_O (20?ml) was stirred until a clear solution was obtained. On slow evaporation of the solvent colourless plate crystals of the title compand suitable for X-ray analysis were obtained accidently.

The dielectric constant of the title compound as a function of temperature goes smoothly between 93 and 363?K and there is no dielectric anomaly observed within the measured temperature range.

### Refinement

The C—H H atoms were positioned with idealized geometry and refined using a riding model (*U*_iso_(H) = 1.2 *U*_eq_(C). The O—H H atoms were located in difference map, their bond lengths set to ideal values and finally they were refined using a riding model (*U*_iso_(H) = 1.5 *U*_eq_(O). Two carbon atoms are disordered and were refined using a split model and sof of 0.7 and 0.3. The C atoms with lower occupancy were refined only isotropic. The absolute structure was determined on the basis of 1134 Friedel-pairs.

### Crystal data

**Table:**

C_8_H_14_N_3_^+^·Br^−^·2H_2_O	*F*(000) = 552
*M**_r_* = 268.16	*D*_x_ = 1.502 Mg m^−^^3^
Orthorhombic, *P*2_1_2_1_2_1_	Mo *K*α radiation, λ = 0.71073 Å
Hall symbol: P 2ac 2ab	Cell parameters from 3350 reflections
*a* = 7.461 (5) Å	θ = 6.3–55.2°
*b* = 12.008 (7) Å	µ = 3.45 mm^−^^1^
*c* = 13.236 (8) Å	*T* = 293 K
*V* = 1185.8 (13) Å^3^	Prism, colourless
*Z* = 4	0.20 × 0.20 × 0.20 mm

### Data collection

**Table:**

Rigaku Mercury CCD diffractometer	2711 independent reflections
Radiation source: fine-focus sealed tube	2219 reflections with *I* > 2σ(*I*)
graphite	*R*_int_ = 0.073
Detector resolution: 13.6620 pixels mm^-1^	θ_max_ = 27.5°, θ_min_ = 3.1°
ω scans	*h* = −9→9
Absorption correction: multi-scan (*CrystalClear*; Rigaku, 2005)	*k* = −15→15
*T*_min_ = 0.701, *T*_max_ = 1.000	*l* = −17→17
13047 measured reflections	

### Refinement

**Table:**

Refinement on *F*^2^	Hydrogen site location: inferred from neighbouring sites
Least-squares matrix: full	H-atom parameters constrained
*R*[*F*^2^ > 2σ(*F*^2^)] = 0.041	*w* = 1/[σ^2^(*F*_o_^2^) + (0.0275*P*)^2^] where *P* = (*F*_o_^2^ + 2*F*_c_^2^)/3
*wR*(*F*^2^) = 0.077	(Δ/σ)_max_ < 0.001
*S* = 1.01	Δρ_max_ = 0.29 e Å^−^^3^
2711 reflections	Δρ_min_ = −0.29 e Å^−^^3^
140 parameters	Extinction correction: *SHELXL97* (Sheldrick, 2008), Fc^*^=kFc[1+0.001xFc^2^λ^3^/sin(2θ)]^-1/4^
101 restraints	Extinction coefficient: 0.0055 (11)
Primary atom site location: structure-invariant direct methods	Absolute structure: Flack (1983), 1134 Friedel pairs
Secondary atom site location: difference Fourier map	Flack parameter: 0.033 (14)

### Special details

**Table:**

Geometry. All e.s.d.'s (except the e.s.d. in the dihedral angle between two l.s. planes) are estimated using the full covariance matrix. The cell e.s.d.'s are taken into account individually in the estimation of e.s.d.'s in distances, angles and torsion angles; correlations between e.s.d.'s in cell parameters are only used when they are defined by crystal symmetry. An approximate (isotropic) treatment of cell e.s.d.'s is used for estimating e.s.d.'s involving l.s. planes.
Refinement. Refinement of *F*^2^ against ALL reflections. The weighted *R*-factor *wR* and goodness of fit *S* are based on *F*^2^, conventional *R*-factors *R* are based on *F*, with *F* set to zero for negative *F*^2^. The threshold expression of *F*^2^ > σ(*F*^2^) is used only for calculating *R*-factors(gt) *etc*. and is not relevant to the choice of reflections for refinement. *R*-factors based on *F*^2^ are statistically about twice as large as those based on *F*, and *R*- factors based on ALL data will be even larger.

### Fractional atomic coordinates and isotropic or equivalent isotropic displacement parameters (Å^2^)

**Table:**

	*x*	*y*	*z*	*U*_iso_*/*U*_eq_	Occ. (<1)
N1	0.3080 (4)	0.2169 (2)	0.79551 (19)	0.0332 (7)	
C1	0.3169 (6)	0.2800 (3)	0.8942 (3)	0.0470 (10)	
H1A	0.3725	0.2343	0.9458	0.056*	
H1B	0.1972	0.2997	0.9165	0.056*	
C2	0.4287 (6)	0.3857 (3)	0.8761 (3)	0.0623 (11)	
H2A	0.3617	0.4499	0.8995	0.075*	
H2B	0.5381	0.3812	0.9154	0.075*	
C3	0.2110 (6)	0.2873 (3)	0.7178 (3)	0.0640 (13)	
H3A	0.0839	0.2899	0.7330	0.077*	0.70
H3B	0.2262	0.2556	0.6509	0.077*	0.70
H3C	0.1046	0.3208	0.7470	0.077*	0.30
H3D	0.1755	0.2422	0.6604	0.077*	0.30
C4	0.2926 (10)	0.4068 (6)	0.7216 (5)	0.053 (2)	0.70
H4A	0.3034	0.4364	0.6536	0.063*	0.70
H4B	0.2148	0.4557	0.7600	0.063*	0.70
C4'	0.349 (2)	0.3801 (15)	0.6842 (12)	0.078 (8)	0.30
H4C	0.4151	0.3555	0.6253	0.093*	0.30
H4D	0.2852	0.4480	0.6669	0.093*	0.30
C5	0.4948 (6)	0.1953 (3)	0.7609 (4)	0.0645 (12)	
H5A	0.4948	0.1434	0.7048	0.077*	0.70
H5B	0.5654	0.1638	0.8154	0.077*	0.70
H5C	0.4924	0.1661	0.6926	0.077*	0.30
H5D	0.5497	0.1398	0.8042	0.077*	0.30
C6	0.5746 (11)	0.3096 (5)	0.7274 (8)	0.0645 (12)	0.70
H6A	0.6987	0.3144	0.7489	0.077*	0.70
H6B	0.5718	0.3148	0.6543	0.077*	0.70
C6'	0.6115 (17)	0.3066 (10)	0.7634 (12)	0.078 (8)	0.30
H6C	0.6908	0.3071	0.8215	0.094*	0.30
H6D	0.6830	0.3135	0.7025	0.094*	0.30
N2	0.4744 (4)	0.4012 (3)	0.7705 (3)	0.0493 (8)	
C7	0.2164 (5)	0.1068 (3)	0.8091 (3)	0.0435 (9)	
H7A	0.2139	0.0676	0.7450	0.052*	
H7B	0.2836	0.0619	0.8568	0.052*	
C8	0.0335 (6)	0.1213 (3)	0.8459 (3)	0.0488 (10)	
N3	−0.1082 (6)	0.1335 (3)	0.8753 (3)	0.0724 (11)	
Br1	0.08721 (5)	−0.01932 (3)	0.56114 (3)	0.05169 (15)	
O1	0.4108 (4)	0.1366 (2)	0.4565 (2)	0.0779 (9)	
H1O1	0.3519	0.0980	0.4953	0.117*	
H2O1	0.4970	0.1418	0.4946	0.117*	
O2	0.2440 (4)	0.3445 (2)	0.4497 (2)	0.0736 (9)	
H1O2	0.2887	0.2823	0.4448	0.110*	
H2O2	0.3207	0.3932	0.4458	0.110*	

### Atomic displacement parameters (Å^2^)

**Table:**

	*U*^11^	*U*^22^	*U*^33^	*U*^12^	*U*^13^	*U*^23^
N1	0.0371 (15)	0.0258 (15)	0.0368 (16)	0.0016 (13)	−0.0012 (12)	−0.0057 (12)
C1	0.061 (3)	0.042 (2)	0.038 (2)	−0.0100 (19)	0.0000 (18)	−0.0081 (16)
C2	0.063 (3)	0.049 (2)	0.074 (3)	−0.022 (3)	0.012 (3)	−0.017 (2)
C3	0.079 (3)	0.056 (3)	0.057 (2)	−0.013 (3)	−0.027 (2)	0.021 (2)
C4	0.070 (4)	0.044 (4)	0.044 (4)	0.010 (3)	0.003 (3)	0.012 (3)
C4'	0.12 (2)	0.036 (10)	0.081 (15)	−0.034 (12)	−0.028 (13)	0.018 (10)
C5	0.051 (2)	0.041 (2)	0.101 (3)	0.0023 (19)	0.032 (2)	−0.009 (2)
C6	0.051 (2)	0.041 (2)	0.101 (3)	0.0023 (19)	0.032 (2)	−0.009 (2)
C6'	0.076 (13)	0.085 (13)	0.072 (13)	−0.067 (12)	0.050 (10)	−0.046 (10)
N2	0.0519 (19)	0.0353 (17)	0.061 (2)	−0.0021 (15)	0.0030 (16)	0.0087 (16)
C7	0.051 (2)	0.0298 (19)	0.049 (2)	−0.0041 (18)	0.0025 (19)	0.0010 (16)
C8	0.052 (3)	0.042 (2)	0.052 (2)	−0.0124 (19)	−0.0037 (19)	−0.0013 (18)
N3	0.058 (3)	0.072 (3)	0.087 (3)	−0.014 (2)	0.004 (2)	−0.012 (2)
Br1	0.0441 (2)	0.0516 (2)	0.0595 (2)	0.00308 (19)	−0.00010 (19)	−0.00776 (18)
O1	0.0656 (19)	0.0689 (19)	0.099 (2)	0.0058 (19)	−0.008 (2)	0.0238 (17)
O2	0.0561 (18)	0.0486 (17)	0.116 (3)	−0.0053 (15)	−0.009 (2)	0.0014 (18)

### Geometric parameters (Å, °)

**Table:**

N1—C5	1.490 (5)	C4'—H4D	0.9700
N1—C7	1.499 (4)	C5—C6	1.560 (8)
N1—C1	1.511 (4)	C5—C6'	1.595 (12)
N1—C3	1.515 (5)	C5—H5A	0.9700
C1—C2	1.538 (5)	C5—H5B	0.9700
C1—H1A	0.9700	C5—H5C	0.9700
C1—H1B	0.9700	C5—H5D	0.9700
C2—N2	1.451 (5)	C6—N2	1.448 (7)
C2—H2A	0.9700	C6—H6A	0.9700
C2—H2B	0.9700	C6—H6B	0.9700
C3—C4	1.560 (8)	C6'—N2	1.532 (13)
C3—C4'	1.579 (14)	C6'—H6C	0.9700
C3—H3A	0.9700	C6'—H6D	0.9700
C3—H3B	0.9700	C7—C8	1.459 (6)
C3—H3C	0.9700	C7—H7A	0.9700
C3—H3D	0.9700	C7—H7B	0.9700
C4—N2	1.504 (8)	C8—N3	1.136 (5)
C4—H4A	0.9700	O1—H1O1	0.8201
C4—H4B	0.9700	O1—H2O1	0.8200
C4'—N2	1.500 (14)	O2—H1O2	0.8201
C4'—H4C	0.9700	O2—H2O2	0.8200
			
C5—N1—C7	108.0 (3)	N1—C5—C6'	111.0 (5)
C5—N1—C1	108.2 (3)	C6—C5—C6'	20.1 (9)
C7—N1—C1	111.0 (3)	N1—C5—H5A	110.3
C5—N1—C3	109.6 (3)	C6—C5—H5A	110.3
C7—N1—C3	110.8 (3)	C6'—C5—H5A	123.7
C1—N1—C3	109.2 (3)	N1—C5—H5B	110.3
N1—C1—C2	107.6 (3)	C6—C5—H5B	110.3
N1—C1—H1A	110.2	C6'—C5—H5B	90.9
C2—C1—H1A	110.2	H5A—C5—H5B	108.6
N1—C1—H1B	110.2	N1—C5—H5C	109.4
C2—C1—H1B	110.2	C6—C5—H5C	93.5
H1A—C1—H1B	108.5	C6'—C5—H5C	109.4
N2—C2—C1	112.6 (3)	H5A—C5—H5C	18.8
N2—C2—H2A	109.1	H5B—C5—H5C	124.1
C1—C2—H2A	109.1	N1—C5—H5D	109.4
N2—C2—H2B	109.1	C6—C5—H5D	127.7
C1—C2—H2B	109.1	C6'—C5—H5D	109.4
H2A—C2—H2B	107.8	H5A—C5—H5D	90.6
N1—C3—C4	107.7 (4)	H5B—C5—H5D	20.4
N1—C3—C4'	105.9 (7)	H5C—C5—H5D	108.0
C4—C3—C4'	26.6 (8)	N2—C6—C5	111.1 (5)
N1—C3—H3A	110.2	N2—C6—H6A	109.4
C4—C3—H3A	110.2	C5—C6—H6A	109.4
C4'—C3—H3A	132.1	N2—C6—H6B	109.4
N1—C3—H3B	110.2	C5—C6—H6B	109.4
C4—C3—H3B	110.2	H6A—C6—H6B	108.0
C4'—C3—H3B	86.8	N2—C6'—C5	104.9 (8)
H3A—C3—H3B	108.5	N2—C6'—H6C	110.8
N1—C3—H3C	110.6	C5—C6'—H6C	110.8
C4—C3—H3C	85.7	N2—C6'—H6D	110.8
C4'—C3—H3C	110.6	C5—C6'—H6D	110.8
H3A—C3—H3C	26.3	H6C—C6'—H6D	108.8
H3B—C3—H3C	128.5	C6—N2—C2	113.8 (5)
N1—C3—H3D	110.6	C6—N2—C4'	84.0 (10)
C4—C3—H3D	130.2	C2—N2—C4'	124.4 (7)
C4'—C3—H3D	110.6	C6—N2—C4	109.2 (5)
H3A—C3—H3D	85.1	C2—N2—C4	102.0 (4)
H3B—C3—H3D	25.6	C4'—N2—C4	27.8 (9)
H3C—C3—H3D	108.7	C6—N2—C6'	21.1 (8)
N2—C4—C3	109.0 (5)	C2—N2—C6'	96.9 (6)
N2—C4—H4A	109.9	C4'—N2—C6'	104.3 (10)
C3—C4—H4A	109.9	C4—N2—C6'	127.5 (6)
N2—C4—H4B	109.9	C8—C7—N1	111.2 (3)
C3—C4—H4B	109.9	C8—C7—H7A	109.4
H4A—C4—H4B	108.3	N1—C7—H7A	109.4
N2—C4'—C3	108.2 (9)	C8—C7—H7B	109.4
N2—C4'—H4C	110.1	N1—C7—H7B	109.4
C3—C4'—H4C	110.1	H7A—C7—H7B	108.0
N2—C4'—H4D	110.1	N3—C8—C7	179.2 (5)
C3—C4'—H4D	110.1	H1O1—O1—H2O1	94.5
H4C—C4'—H4D	108.4	H1O2—O2—H2O2	111.1
N1—C5—C6	106.9 (4)		

### Hydrogen-bond geometry (Å, °)

**Table:**

*D*—H···*A*	*D*—H	H···*A*	*D*···*A*	*D*—H···*A*
O1—H1O1···Br1	0.82	2.58	3.354 (3)	159
O2—H1O2···O1	0.82	1.98	2.791 (4)	170
O1—H2O1···O2^i^	0.82	1.99	2.788 (5)	164
O2—H2O2···Br1^i^	0.82	2.50	3.314 (3)	172
C7—H7A···Br1	0.97	2.81	3.740 (5)	161
C7—H7B···Br1^ii^	0.97	2.92	3.792 (8)	151

Symmetry codes: (i) *x*+1/2, −*y*+1/2, −*z*+1; (ii) −*x*+1/2, −*y*, *z*+1/2.
